# Association Between Serum Aminotransferases and Risk of New-Onset Cardiometabolic Disease in a Healthy Chinese Population: A Cohort Study

**DOI:** 10.3389/fpubh.2022.902393

**Published:** 2022-06-09

**Authors:** Qin Lan, Yuming Zhang, Fang Lin, Qingshu Meng, Nicholas Jan Buys, Huimin Fan, Jing Sun

**Affiliations:** ^1^Shanghai East Hospital, Tongji University, Shanghai, China; ^2^School of Medicine, Tongji University, Shanghai, China; ^3^School of Medicine and Dentistry, Griffith University, Gold Coast, QLD, Australia; ^4^Menzies Health Institute Queensland, Griffith University, Gold Coast, QLD, Australia

**Keywords:** aspartate aminotransferase, alanine aminotransferase, diabetes mellitus, cohort study, metabolic syndrome, risk factor

## Abstract

**Purpose:**

This study aimed to investigate the association between serum aspartate aminotransferase (AST), alanine aminotransferase (ALT), and incident metabolic disease in a cohort of community-based older Chinese people.

**Patients and Methods:**

Five thousand healthy Gaohang residents who attended community health checks at the Shanghai East Hospital in 2013 were recruited. Biological, biochemical, and lifestyle variables were collected. The cohort was followed for new-onset metabolic disease in 2014 and 2017, with a final study population of 3,123 (63%) after follow-up. The study outcome included type-2 diabetes mellitus and metabolic syndrome.

**Results:**

Baseline AST and ALT were associated with incident type-2 diabetes mellitus (HR 1.019, 95% CI 1.006–1.032, *p* = 0.003 and HR 1.016, 95% CI 1.008–1.025, *p* < 0.001 respectively). These associations persisted after adjusting for traditional risk factors including age, sex, income, waist circumference, systolic blood pressure, diastolic blood pressure, HbA1c, triglyceride, cholesterol, HDL and eGFR. Baseline AST and ALT were associated with incident metabolic syndrome in the crude analysis (HR 0.980, 95% CI 0.965–0.996, *p* = 0.012 and HR 0.992, 95% CI 0.988–0.997, *p* = 0.001, respectively). However, the association between AST and ALT with metabolic syndrome was non-significant after adjusting for biochemical parameters such as the lipid profile.

**Conclusion:**

This study demonstrated that serum AST and ALT are associated with new-onset type-2 diabetes mellitus, independent of traditional risk factors, in a cohort of older Chinese people. These findings may contribute to disease risk stratification and management in type-2 diabetes.

## Introduction

Cardiometabolic disease is an umbrella term given to a wide range of cardiovascular and metabolic diseases, typically including ischemic heart disease, type-2 diabetes mellitus (T2DM), lipid disorders, and chronic renal failure (1–3). As the global burden of chronic diseases rose, there have been parallel increases in the incidence and prevalence of cardiometabolic disease (4). In 2016, an estimated 17.9 and 1.6 million deaths were attributed to cardiovascular diseases and diabetes alone (5). In China, the prevalence of cardiometabolic disease morbidity in the general population have more than doubled between 2009 and 2015 (6). This increase is especially pronounced in the older population (7, 8), potentially due to increased frailty and the number of risk factors such as hypertension, dyslipidemia, and inflammation (7–10). Cardiometabolic disease in the aging population is an urgent issue, as it increases the risk of life-limiting comorbidities (11).

Elevated liver enzymes such as aspartate aminotransferase (AST) and alanine aminotransferase (ALT) are traditionally interpreted as markers of liver dysfunction, such as in non-alcoholic fatty liver disease (NAFLD) (12, 13). However, as AST is also expressed in other tissues including the heart and kidneys, elevated levels of AST in the circulation can also indicate cardiovascular pathologies (13). Previous studies on various Western cohorts have identified associations between serum aminotransferases and numerous cardiovascular and metabolic diseases such as atrial fibrillation (14), coronary heart disease (15, 16), ischemic stroke (16), cardiovascular mortality (17), as well as diabetes (18). Further associations have been reported between elevated aminotransferases and established risk factors of cardiometabolic disease, including high blood pressure, body mass index (BMI), and fasting plasma glucose (19, 20). However, conflicting findings have also been reported (21). Such heterogeneity suggests individual liver enzymes may have differential impacts on cardiometabolic risk, as well as possible ethnic variation. Current gaps exist in establishing the relationship between hepatic aminotransferases and cardiometabolic risk in previously unexamined populations. To the best of the author's knowledge, there are limited report of the long-term effects of aminotransferases on cardiometabolic disease development in the community-based older population of China.

This study attempts to address this gap by investigating the relationship between serum AST, ALT, and 4-year incidence of cardiometabolic disease, including diabetes mellitus and metabolic syndrome in a community-based cohort of Gaohang residents. It is hypothesized that higher AST and ALT act as predictors of metabolic disease outcome.

## Materials and Methods

### Study Design and Population

**Figure 1:**
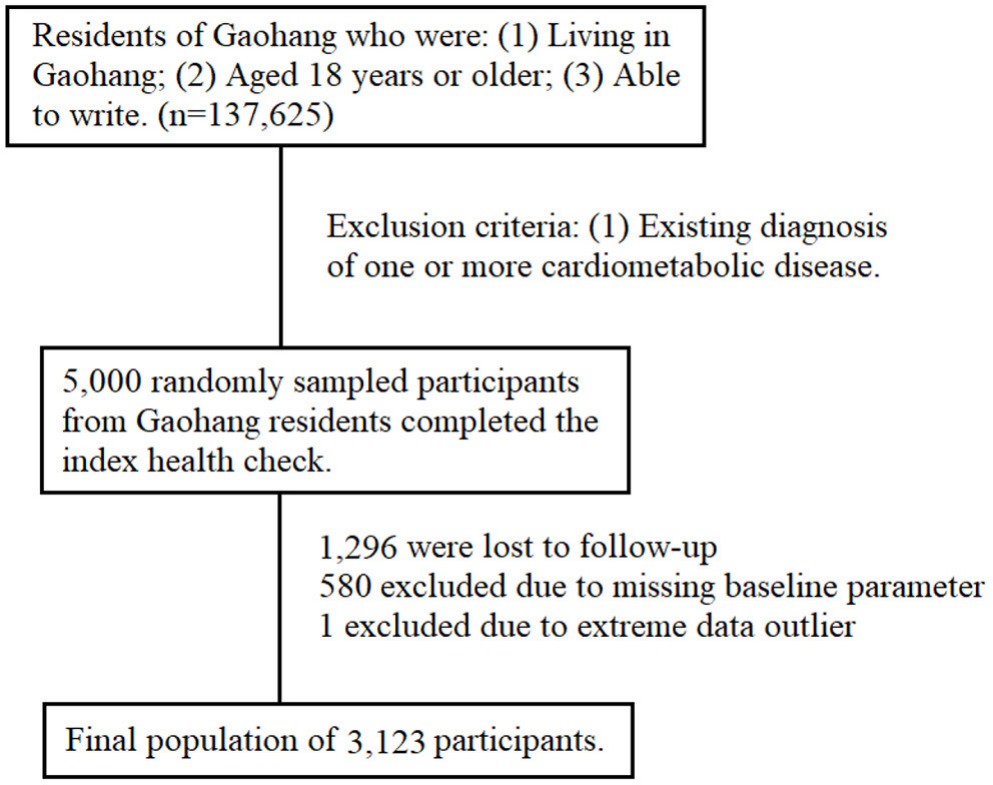
Flow-chart of participant selection process.

The study population included 5,000 healthy individuals who were randomly sampled from a total population of 137,625 residents of the Gaohang community, in the Pudong district of Shanghai city. The sampling process is outlined in Figure 1. The inclusion criteria were: (1) currently living in Gaohang; (2) aged 18 years or over; and (3) able to write to provide consent. Persons with one or more established cardiometabolic disease at baseline were excluded. At the 4-year follow-up, 1,296 participants were lost to follow-up with unascertained disease status. A further 581 were excluded from analysis due to missing baseline parameter or being an outlier. The final population consisted of 3,123 (63%) individuals. The pattern of missing data suggests no preferential loss of individual variables. Analyses between those who were lost to follow-up and those who were retained showed no significant difference in baseline AST, ALT, and other key covariates. Comparison against analysis with multiple imputation shows no significant difference from complete case analysis. All information was utilized with informed consent, and approved by the Ethics Committee of Shanghai East Hospital.

### Data Collection

Biological, biochemical and behavioral data were collected through standardized health checks at study commencement and follow-ups. The index health check included biological measurements of height, weight, waist circumference, and systolic blood pressure (SBP) and diastolic blood pressure (DBP). Resting blood pressure was taken manually with a mercury sphygmomanometer after 5 min of rest and averaged from three measurements. Single blood samples were taken after an overnight fast of 10 h and stored at −80°C before undergoing double-blind analysis at the Shanghai East Hospital using standardized automated methods. All measurements were repeated at the follow-up checks in 2014 and 2017. Reference ranges for blood tests were defined per the Chinese Health Industry Guideline (22). Health-related behaviors and past medical history were collected using interviews and standardized questionnaires, including smoking, alcohol drinking, and exercise habits. Smoking status was categorized into non-smoker, current smoker, and ex-smoker. Alcohol drinking was defined as >50 g for more than a year, and categorized into non-drinker, current drinker, and ex-drinker. Exercise status was defined and categorized using the Physical Exercise Guideline into sedentary, light-intensity exercise, and moderate-intensity exercise (23).

### Study Outcome

Diabetes mellitus was defined based on parameters established by the Chinese Diabetes Society, namely fasting glucose >7.0 mmol/L (24). Metabolic syndrome was defined using parameters established by the revised National Cholesterol Education Program Adult Treatment panel III (NCEP ATP III) criteria, with population-specific adjustments made to waist circumference (≥90 cm in Asian men, ≥80 cm in Asian women) (25).

To assess the study outcomes, participants were followed from the index health check in 2013 and censored at the occurrence of cardiometabolic disease, death, or the last follow-up in 2017, whichever came first. Diagnoses were confirmed with hospital records where relevant.

### Statistical Analysis

Statistical analysis was performed using SPSS® 26.0 (SPSS, Chicago, IL, USA), with statistical significance set at *P* < 0.05.

Categorical variables were expressed as numbers and percentages, while continuous variables were expressed as mean and standard deviation. ANOVA test and χ^2^ test were performed on continuous and categorical variables, respectively, to identify covariates. Extreme outliers were excluded from analysis. Benjamin-Hochberg test was used to adjust for false discovery rate across multiple comparisons.

The association between baseline aminotransferase concentrations and incident metabolic disease was assessed using Cox regression analysis, using raw baseline AST and ALT as continuous variables. Assumption testing was conducted prior to analysis. The proportional hazards assumption was tested using scaled Schoenfeld residuals. Variables not satisfying the assumption of equal proportion were entered as time-dependent. Influential observations were identified using the dfbeta approach and extreme outliers were removed. Linearity was assessed graphically by plotting Martingale residuals against fitted values. Collinearity was assessed using the Farrar-Glauber test. Fasting glucose and BMI were subsequently removed from the multivariate model given their significant correlation with HbA1c and waist circumference, respectively. Four models are presented in the analysis. Model 1 presents the unadjusted crude hazard ratio. Model 2 is adjusted for age, sex, and income status. Model 3 further accounts for biological measurements including waist circumference, SBP and DBP. Model 4 adjusts for Model 3 and biochemical measurements including HbA1c, triglyceride, cholesterol, and eGFR. The hazard ratio (HR), 95% confidence interval (CI), and *p*-value are presented for each Model across Type-2 diabetes mellitus and metabolic syndrome.

## Results

### Population Characteristics

The baseline population characteristics by disease status at follow-up are presented in Table 1. Monthly income was significantly associated with disease outcome (*p* = 0.004). Healthy participants who did not develop metabolic disease was more likely to earn lower income (28.2%) compared to those who developed disease (22.4%). Compared to non-disease participants, those who developed disease were more likely to work in an office (17.7 vs. 14.7%), and less likely to work in farming (41.0 vs. 43.7%), however this did not reach statistical significance (*p* = 0.184). Similarly, no significant differences were identified across gender, education, smoking status, alcohol drinking, and exercise status.

**Table 1:** Demographic variables associated with cardiometabolic disease.

	**Metabolic disease outcome**		
	**Non-diseased**	**Diseased**	***P***
	**(*n* = 2,554)**	**(*n* = 569)**	
Categorical variables *N* (%)			
Male sex	1097 (43.0)	239 (42.0)	0.679
Occupation			
Office worker	374 (14.7)	100 (17.7)	0.184
Operator	911 (35.7)	194 (34.3)	
Farmer	1116 (43.7)	232 (41.0)	
Others	151 (5.9)	40 (7.1)	
Education			
Primary or below	504 (19.8)	101 (17.8)	0.085
Primary	563 (22.1)	109 (19.2)	
Secondary	1252 (49.1)	290 (51.1)	
Tertiary or above	232 (9.1)	67 (11.8)	
Monthly income^a^			
<1,500	710 (28.2)	125 (22.4)	**0.004**
1,500–2,000	463 (18.4)	87 (15.6)	
2,000–2,500	575 (22.8)	149 (26.8)	
>2,500	773 (30.7)	196 (35.2)	
Smoking status			
Non-smoker	1973 (77.3)	442 (77.7)	0.632
Smoker	351 (13.7)	71 (12.5)	
Ex-smoker	230 (9.0)	56 (9.8)	
Alcohol drinking			
Non-drinker	2106 (82.5)	478 (84.0)	0.620
Drinker^b^	99 (3.9)	22 (3.9)	
Ex-drinker	349 (13.7)	69 (12.1)	
Exercise status			
Sedentary	119 (4.9)	23 (4.2)	0.669
Light-intensity	1689 (69.0)	370 (68.1)	
Moderate-intensity	639 (26.1)	150 (27.6)	

*Values expressed as n (%).*

*^a^All monetary units in Chinese Yuan (CNY).*

*^b^Includes low, moderate, and high levels.*

*p <0.05 used and statistically significant results bolded*.

The biochemical variables associated with disease status at follow-up is shown in Table 2. Compared to non-diseased participants, those who developed diabetes had higher baseline AST (*p* = 0.044), ALT (*p* = 0.011), BMI (*p* = 0.002), and triglyceride (*p* = 0.002). Lower baseline fasting glucose (*p* = 0.006) and HDL (*p* = 0.001) were noted. Metabolic syndrome was associated with lower baseline AST (*p* = 0.005), ALT (*p* < 0.001), age (*p* < 0.001), BMI (*p* < 0.001), waist circumference (*p* < 0.001), SBP (*p* < 0.001), DBP (*p* < 0.001), and triglyceride (*p* = 0.020).

**Table 2:** Biochemical variables associated with disease status at follow-up.

**Biochemical variables: M(SD)**	**Diabetes mellitus**			**Metabolic syndrome**		
	**Non-diseased (*n* = 2,901)**	**Diseased (*n* = 222)**	***P***	**Non-diseased (*n* = 2,737)**	**Diseased (*n* = 386)**	***P***
Serum AST at baseline (IU/L)	21.9 (8.3)	23.1 (11.2)	**0.044** ^a^	22.2 (8.6)	20.7 (6.1)	**0.005** ^a^
Serum ALT at baseline (IU/L)	18.2 (11.3)	20.8 (15.2)	**0.011** ^a^	18.6 (12.0)	16.6 (9.0)	**<0.001** ^a^
Age (years)	71.6 (6.4)	71.0 (5.6)	0.133	71.7 (9.0)	70.5 (5.9)	**<0.001**
BMI (kg/m^2^)	24.6 (3.4)	25.2 (3.0)	**0.002**	24.7 (3.5)	23.8 (2.6)	**<0.001**
Waist circumference (cm)	86.4 (9.2)	87.5 (7.9)	0.052	87.1 (9.1)	82.5 (7.9)	**<0.001**
SBP (mmHg)	138.6 (17.1)	140.1 (16.8)	0.202	139.6 (16.9)	132.6 (17.2)	**<0.001**
DBP (mmHg)	81.8 (8.7)	81.9 (8.3)	0.782	82.1 (8.7)	79.9 (8.0)	**<0.001**
Fasting glucose (mmol/L)	5.7 (1.9)	5.5 (0.7)	**0.006** ^**a**^	5.7 (1.8)	5.6 (1.7)	0.517^a^
HbA1c (%)	6.3 (1.1)	6.0 (0.3)	0.958^a^	6.3 (1.0)	4.3 (1.1)	0.800^a^
TG (mmol/L)	1.6 (1.1)	1.8 (1.2)	**0.002** ^**a**^	1.7 (1.2)	1.5 (0.7)	**0.020** ^**a**^
TC (mmol/L)	5.0 (1.0)	4.9 (0.9)	0.390	5.0 (1.0)	4.9 (0.9)	0.269
HDL (mmol/L)	1.5 (0.4)	1.4 (0.3)	**0.001**	1.5 (0.4)	1.5 (0.4)	0.880
LDL (mmol/L)	3.3 (0.9)	3.3 (0.9)	0.837	3.3 (0.9)	3.3 (0.8)	0.624
eGFR (mL/min/1.73 m^2^)	78.5 (40.0)	79.9 (38.0)	0.446^a^	78.1 (39.0)	82.5 (44.9)	0.098^a^

*Values expressed as M(SD). SBP, systolic blood pressure; DBP, diastolic blood pressure; TG, triglyceride; TC, total cholesterol; HDL, high density lipoprotein; LDL, low density lipoprotein.*

*^a^Mann-Whitney test used to calculate P-value for non-parametric variables.*

*p <0.05 used and statistically significant results bolded*.

### Biochemical Factors Associated With Baseline Aminotransferase

The association between baseline serum aminotransferase and other biochemical parameters is shown in Table 3. AST was highly correlated with serum ALT (*r* = 0.631, *p* < 0.001). However, its correlation to other biochemical variables was low. Slight positive correlations were identified between AST and DBP (*r* = 0.059, *p* < 0.001), HDL (*r* = 0.068, *p* < 0.001), cholesterol (*r* = 0.054, *p* = 0.001), BMI (*r* = 0.042, *p* = 0.009), triglyceride (*r* = 0.041, *p* = 0.009), waist circumference (*r* = 0.037, *p* = 0.020), and SBP (*r* = 0.035, *p* = 0.026). Baseline eGFR was negatively correlated with AST (*r* = −0.293, *p* < 0.001).

**Table 3:** Correlation between biochemical variables and baseline AST and ALT.

**Biochemical variable**	**Baseline AST (IU/L)**		**Baseline ALT (IU/L)**	
	***R***	***P***	***R***	***P***
Serum AST at baseline (IU/L)	1	-	0.631	**<0.001**
Serum ALT at baseline (IU/L)	0.631	**<0.001**	1	-
Age (years)	0.012	0.456	−0.166	**<0.001**
BMI (kg/m^2^)	0.042	**0.009**	0.251	**<0.001**
Waist circumference (cm)	0.037	**0.020**	0.218	**<0.001**
SBP (mmHg)	0.035	**0.026**	0.062	**<0.001**
DBP (mmHg)	0.059	**<0.001**	0.106	**<0.001**
Fasting glucose (mmol/L)	0.009	0.584	0.194	**<0.001**
HbA1c (%)	−0.025	0.122	0.127	**<0.001**
TG (mmol/L)	0.041	**0.009**	0.214	**<0.001**
TC (mmol/L)	0.054	**0.001**	0.029	0.066
HDL (mmol/L)	0.068	**<0.001**	−0.144	**<0.001**
LDL (mmol/L)	−0.008	0.599	0.006	0.713
eGFR (mL/min/1.73m^2^)	−0.293	**<0.001**	−0.133	**<0.001**

*Values expressed as M(SD). SBP, systolic blood pressure; DBP, diastolic blood pressure; TG, triglyceride; TC, total cholesterol; HDL, high density lipoprotein; LDL, low density lipoprotein.*

*p <0.05 used and statistically significant results bolded*.

Baseline ALT was positively correlated with BMI (*r* = 0.251, *p* < 0.001), waist circumference (*r* = 0.218, *p* < 0.001), and triglyceride (*r* = 0.214, *p* < 0.001) at low correlation level, and shows slight correlations with SBP (*r* = 0.062, *p* < 0.001), DBP (*r* = 0.106, *p* < 0.001), fasting glucose (*r* = 0.194, *p* < 0.001), HbA1c (*r* = 0.127, *p* < 0.001). Slight negative correlations were present between ALT and age (*r* = −0.166, *p* < 0.001), HDL (*r* = −0.144, *p* < 0.001), and eGFR (*r* = −0.133, *p* < 0.001).

As these risk factors had low correlation with AST, factors including DBP, HDL, cholesterol, triglyceride, waist circumference, SBP, and eGFR will be controlled for in the Cox regression model when assessing the association between AST and metabolic disease. Likewise, biochemical variables associated with ALT will be adjusted for in the Cox regression model, waist circumference, triglyceride, SBP, DBP, fasting glucose HbA1c; very low and negative correlation with age, HDL and eGFR, BMI, waist circumference, triglyceride, SBP, DBP, fasting glucose HbA1c, age, HDL, and eGFR will be controlled for in the Cox regression model when assessing the association between ALT and metabolic disease.

### Aminotransferases and New-Onset Cardiometabolic Disease

**Figure 2:**
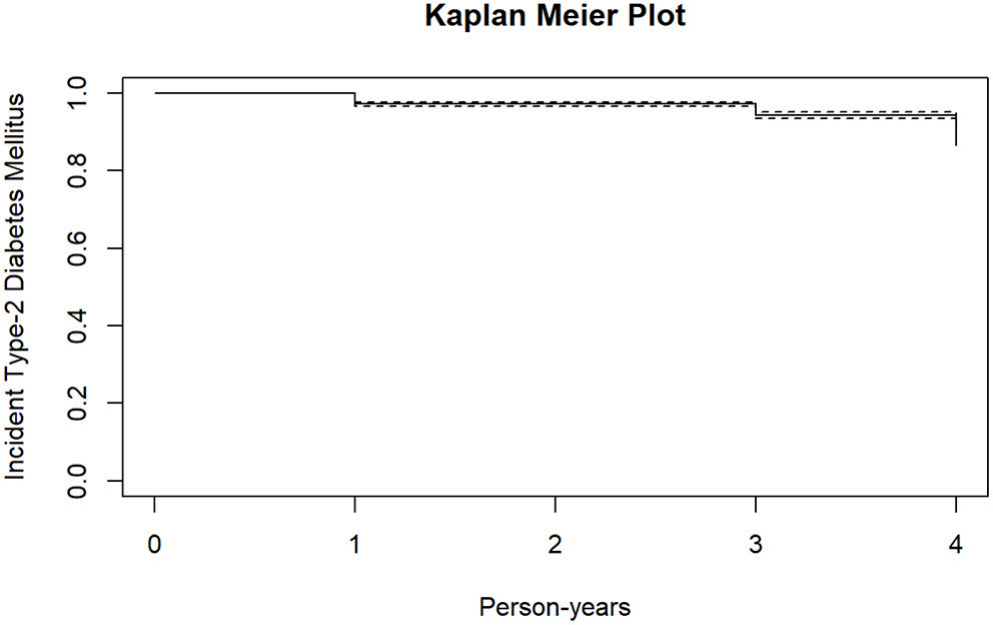
Kaplan meier plot for incident type-2 diabetes mellitus.

**Figure 3:**
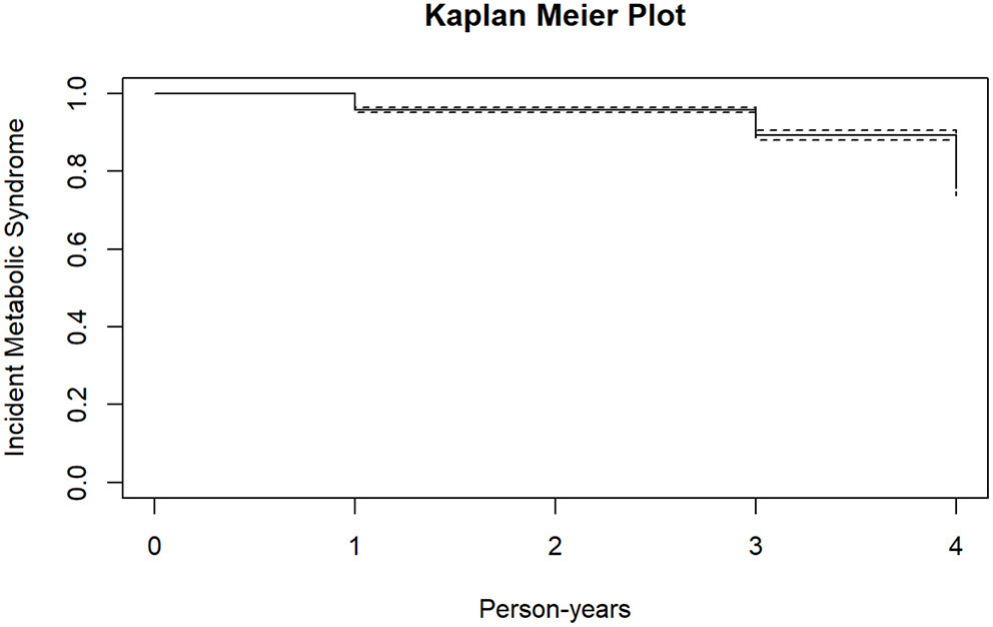
Kaplan meier plot for incident metabolic syndrome.

Figures 2, 3 shows the survival curves for incident type-2 diabetes mellitus and metabolic syndrome. At follow-up, 569 (18.2%) participants developed at least one metabolic outcome. A total of 222 participants developed incident type-2 diabetes mellitus while 386 participants developed incident metabolic syndrome.

Table 4 demonstrates the association between baseline aminotransferases and the incidence of metabolic disease.

**Table 4:** Risk of cardiometabolic disease by baseline AST and ALT.

**Disease status**	**Baseline AST (IU/L)**		**Baseline ALT (IU/L)**	
	**HR (95% CI)**	***P***	**HR (95% CI)**	***P***
**Diabetes mellitus**				
Model 1	1.018 (1.005–1.030)	**0.005**	1.016 (1.008–1.024)	**<0.001**
Model 2	1.018 (1.005–1.030)	**0.005**	1.016 (1.009–1.025)	**<0.001**
Model 3	1.017 (1.004–1.029)	**0.008**	1.015 (1.007–1.023)	**<0.001**
Model 4	1.019 (1.006–1.032)	**0.003**	1.016 (1.008–1.025)	**<0.001**
**Metabolic syndrome**				
Model 1	0.980 (0.965–0.996)	**0.012**	0.992 (0.988–0.997)	**0.001**
Model 2	0.980 (0.965–0.996)	**0.014**	0.992 (0.987–0.996)	**<0.001**
Model 3	0.982 (0.966–0.998)	**0.026**	0.995 (0.991–0.999)	**0.029**
Model 4	0.987 (0.971–1.003)	0.116	0.996 (0.992–1.000)	0.075

*HR, hazard ratio; CI, confidence interval.*

*Model 1 is unadjusted.*

*Model 2: adjusted for age, sex and income.*

*Model 3: adjusted for model 2, waist circumference, SBP and DBP.*

*Model 4: adjusted for model 3, HbA1c, triglyceride, cholesterol, HDL and eGFR.*

*p <0.05 used and statistically significant results bolded*.

### Metabolic Syndrome

Baseline AST was associated with lower risk of incident metabolic syndrome in the unadjusted model (HR 0.980, 95% CI 0.965–0.996, *p* = 0.012). This association persisted after adjusting for age, sex, income, waist circumference, SBP and DBP. However, it was attenuated in Model 4 after adjusting for biochemical covariates such as HbA1c, triglyceride, cholesterol and eGFR (*p* = 0.116).

Baseline ALT was associated with a lower risk of metabolic syndrome in Model 1 (HR 0.992, 95% CI 0.988–0.997, *p* = 0.001).Similar to AST, this was also attenuated in Model 4 after adjusting for covariates (*p* = 0.075).

### Diabetes Mellitus

Baseline AST showed a positive association with incident diabetes development (HR 1.019, 95% CI 1.006–1.032, *p* = 0.003), independent of traditional risk factors such as waist circumference, SBP, DBP, HbA1c, triglyceride, cholesterol, and eGFR. This association was significant after adjusting for demographic variables in Model 2 (*p* = 0.005), biological variables in Model 3 (*p* = 0.005), and biochemical variables in Model 4 (*p* = 0.003). ALT also showed a positive association with incident diabetes, with a crude hazard ratio of 1.016 (95% CI 1.008–1.024, *p* < 0.001). The multi-adjusted hazard ratio suggest the association between ALT and incident diabetes mellitus is independent of age, sex, income, waist circumference, SBP, DBP, HbA1c, triglyceride, cholesterol, and eGFR (HR 1.016, 95% CI 1.008–1.025, *p* < 0.001).

## Discussion

### Diabetes Mellitus

Results from this study demonstrated a significant positive association between baseline serum AST, ALT and incident type-2 diabetes mellitus. These associations persisted after adjusting for confounding factors.

Studies across various populations have reported significant associations between high ALT quartiles and incident diabetes, independent of traditional risk factors such as lipid profiles and anthropometric factors (26–29). Our finding is consistent with a previous study on the Chinese population, which reported significant increase in incident diabetes for every log increase in ALT (30).

Previous studies on the association between AST and incident diabetes have reported inconsistent findings on this association. While studies across Asian populations have reported no association between AST and diabetes (26, 27), significant associations have been reported in American populations (18, 31). Similar inconsistencies were observed in the Chinese population (30, 32). Differential inclusion of confounding factors may also have accounted for some heterogeneity as HbA1c was not considered in a number of studies. Given the established relationship between HbA1c and diabetes (26), it is possible that its inclusion would have attenuated the effect of AST in some studies.

Several possible mechanisms have been proposed to explain the association between hepatic aminotransferases and development of diabetes. One explanation is the presence of underlying fatty liver disease, characterized by lipid deposition within hepatocytes (33). The “two-hit” hypothesis argues that a combination of insulin resistance and lipid accumulation leads to fatty infiltration of the liver, which is reflected by increased levels of aminotransferases in the circulation and results in increased risk of diabetes by promoting hyperinsulinaemia and increasing endogenous glucose production (18). Another theory is increased oxidative stress and inflammation secondary to excessive fatty acid deposition, which favors the release of pro-inflammatory adipocytokines and promotes a systemic inflammatory state (34). Chronic low-grade inflammation has consistently been shown to precede the development of diabetes and atherothrombotic diseases, possibly due to its inhibitory effects on insulin signaling (35). However, it is difficult to determine the temporality of this relationship, as elevated aminotransferases may be a result of the inflammation rather than the cause (18). Recent literature further argues that a complex interaction between liver transaminases and other risk factors are responsible, as AST and ALT are known to be associated with additional metabolic risk factors such as abnormal waist circumference, fasting blood glucose, and cholesterol (36), also demonstrated in our study.

### Study Limitations

There are some limitations to this study. First, although multiple covariates were identified and adjusted for, the effect of residual confounding factors may persist. For example, gamma glutamyl-transferase (GGT), alanine phosphatase (ALP) and B6 deficiency were not measured. Second, the true incidence of cardiometabolic disease may be underestimated as the 4-year follow-up period is relatively short for the development of chronic diseases from a healthy population. Last, given the unique demographic profile of the cohort, further studies may be required to confirm the generalizability of findings onto the broader Chinese population.

## Conclusion

Serum AST and ALT were positively associated with 4-year risk of incident type-2 diabetes mellitus among healthy Chinese people, independent of age, gender, income, waist circumference, SBP, DBP, HbA1c, triglyceride, cholesterol and eGFR. A small negative correlation was identified between baseline AST, ALT and incident metabolic syndrome. However, this association was attenuated after adjusting for biochemical variables including HbA1c, triglyceride, cholesterol and eGFR, suggesting lipid profile may be a better predictor of metabolic syndrome rather than AST and ALT. Serum aminotransferases may be a practical tool for identifying high-risk individuals and provide a basis of targeted prevention strategies. However, further studies are required to validate the associations.

## Data Availability Statement

The raw data supporting the conclusions of this article will be made available by the authors, without undue reservation.

## Ethics Statement

The studies involving human participants were reviewed and approved by Ethics Committee of Shanghai East Hospital. The patients/participants provided their written informed consent to participate in this study.

## Author Contributions

JS and HF contributed to the study conception and study design. QL, FL, and QM contributed to the collection of the data. YZ and QL contributed to the data analysis, interpretation of the data, and drafting of the manuscript. JS contributed to the data analysis and review and editing of the manuscript. NB reviewed and edited the manuscript. All authors read and approved the final manuscript.

## Funding

This work was supported by National Nature Science Foundation of China (81670458, 81470393, and 81370434), Shanghai Three year plan on promoting TCM development (ZY(2018-2020)-FWTX-2007), Key Discipline of the Health Industry Project of Pudong Health Bureau of Shanghai (PWZxk2017-01), Three year plan of Pudong Health Bureau of Shanghai (PWYgf2018-05), Three-year plan on TCM of Pudong Health Bureau of Shanghai (PDZY-2018-0603), the National Key Research and Development Program of China (2017YFA0105600), the Science and Technology Commission of Shanghai Municipality (17431906600), and the Health Industry Project of Pudong Health Bureau of Shanghai (No. PW2013E-1).

## Conflict of Interest

The authors declare that the research was conducted in the absence of any commercial or financial relationships that could be construed as a potential conflict of interest.

## Publisher's Note

All claims expressed in this article are solely those of the authors and do not necessarily represent those of their affiliated organizations, or those of the publisher, the editors and the reviewers. Any product that may be evaluated in this article, or claim that may be made by its manufacturer, is not guaranteed or endorsed by the publisher.
